# Recent advances in L-Asparaginase enzyme production and formulation development for acrylamide reduction during food processing

**DOI:** 10.1016/j.fochx.2024.102055

**Published:** 2024-12-02

**Authors:** Arindam Jana, Soumyajit Biswas, Ritu Ghosh, Rahul Modak

**Affiliations:** a Infection and Epigenetics Laboratory, School of Biotechnology, Kalinga Institute of Industrial Technology, Bhubaneswar 751024, Odisha, India; b KIIT - Technology Business Incubator (KIIT-TBI), KIIT-DU, Bhubaneswar 751024, Odisha, India; c University of Tartu: Faculty of Science and Technology, Institute of Technology, Nooruse 1, 50411 Tartu, Estonia

**Keywords:** L-asparaginase, Acrylamide reduction, Enzyme formulation, Acrylamide content in food, Asparaginase activity

## Abstract

L-asparagine is an essential amino acid for cell growth and common constituent of all the proteins. During high temperature food processing it reacts with reducing sugars and leads to acrylamide production through a complex process known as Maillard reaction. L-asparaginase hydrolyses the amine-group of L-asparagine to produce aspartic acid and ammonia. L-asparaginase pre-treatment of potato led to more than 80 % reduction of acrylamide content in foods like french fries, potato chips and in flour-dough based products. New cost-effective strategies for large scale L-asparaginase production and diverse types of formulations will be needed to successfully integrate L-asparaginase in food processing. Here we comprehensively review the recent developments in enzyme production to enhance the yield, activity and specificity of L-asparaginase. Novel liquid and lyophilized formulations are developed to enhance stability and activity of the enzyme under different conditions. These developments present a promising approach to enzymatically mitigate acrylamide formation during food processing.

## Highlights

• L-asparagine in food reacts with sugar to form acrylamide at high temperature.
• L-asparaginase hydrolyzes amino group of asparagine and reduces acrylamide formation in food.
• Novel formulations improve enzyme stability under varied food processing conditions.
• New strategies required to enhance L-asparaginase yield and activity for large-scale production.
• Acrylamide reduction is essential to meet new regulatory standards for safer food.

## 1 Introduction

L-asparaginase (EC 3.5.1.1, ASNase), an enzyme with remarkable therapeutic and industrial potential, has garnered significant attention in recent years. L-asparaginase has been extensively characterized and it is used as injectable drug to treat certain types of acute lymphoblastic leukemia (ALL). It has been tested for other therapeutic applications like treating other types of cancers like, acute myeloblastic leukemia, chronic lymphoma, Hodgkin's disease, autoimmune disorders, antimicrobial agents, collagen induced arthritis (CIA), flesh eating disease, pharyngitis and scarlet fever. Healthcare applications of L-asparaginase has been extensively reviewed by van Trimpont et al. (Van Trimpont et al., 2022), Apart from healthcare, L-asparaginase is used in food industry, biosensor development and in few other applications. High acrylamide content in several commonly consumed processed foods like French fries, potato chips, bread and doughnuts and several others have been reported by U.S. FDA (U.S.FDA, 2006). Acrylamide has been classified as a probable human carcinogen by the International Agency for Research on Cancer (IARC) (IARC, 1994). The acrylamide content in food can be significantly reduced by using L-asparaginase during food processing. Applications of L-asparaginase in diverse food matrices need a wide range of formulation that will help in efficient use of the enzyme. The enzyme has been stabilized by various physical and chemical adsorption methods for different other applications (Nunes et al., 2020), however food applications needs to be comprehensively reviewed for efficient process development and integration with some existing food processing processes to maximize benefits in future. So, the research needs to be expanded more on the unexplored food matrices.

### 1.1 Background on L-asparaginase

L-asparaginase is an enzyme of significant importance due to its diverse applications and impactful properties. Initially identified for its anti-cancer properties, particularly in leukemia treatment (Juluri et al., 2022), which further evolved to find applications in various industries, including healthcare and food production. For different applications, Asparaginase has been isolated from different microorganisms like bacteria, yeast etc. Among bacterial sources, *Escherichia coli* and *Erwinia chrysanthemi* are the most significant due to their high yield, stability, and effectiveness (Ashok et al., 2019). Over last 6 decades several groups reported kinetic properties and catalytic mechanisms of L-asparaginase, but molecular details of enzyme substrate interaction were recently described by Lubkowski et al. (2020). Although these enzymes are significantly diverse in their amino acid sequences but they catalyze the same reaction. *E. coli* L-asparaginase is a homotetrameric protein with native molecular weight of approximately 140 kDa and each monomer has ∼330 amino acids (Lubkowski & Wlodawer, 2021). The enzyme exhibits optimal activity at 37 °C over a pH range of 4.5–11.5. Structurally *E. coli* L-asparaginase enzyme belongs to the class of alpha/beta proteins. Each monomer has two domains with unique topological features (PDB ID 3ECA, 6UOG). The tetramer can be visualized as dimer of two intimate monomers. The active site is formed by the residues from each monomer present in the dimer leading to formation of 2 complete active sites in asparaginase-II. The active site contains several key residues such as Thr12, Thr89, and Asp96 (residue numbering as in *E. coli* L-Asparaginase-II) that are involved in substrate binding and catalysis (Lubkowski & Wlodawer, 2021). Structurally, the active site stays in 2 distinct- ligand free and β-protonated L-Asp bound states (PDB ID 6V23 and 6V25 respectively). Active site is quiet rigid except the highly conserved glycine rich hinge region (Gly10- Gly17) and flexible loop of active site (Asp18- Gly31). Although the hinge and loop region does not interact with the substrate- L-Asn, they may be necessary to accommodate bigger peptides carrying asparagine. L-asparaginase's ability to reduce asparagine level in diverse substrates and its well-characterized biochemical properties makes it the 1st choice in both therapeutics and food industrial application and underscores its multifaceted importance and potential impact on human health and food safety.

### 1.2 Acrylamide formation in processed food

**Fig. 1:**
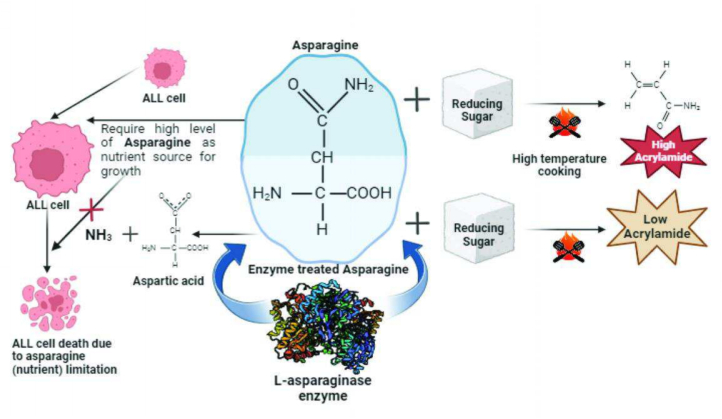
**Schematic summary of role of asparagine and function of L-asparaginase.** Asparagine helps in tumour cell growth and acrylamide formation during high temperature food processing (Maillard reaction). L-asparaginase enzyme hydrolyses the amine group of side chain of asparagine and converts it to aspartic acid, thus reducing the asparagine content in the system. This in turn leads to inhibition of cell growth and reduction in the acrylamide content in food.

During high-temperature cooking processes such as frying, baking and roasting, particularly in carbohydrate-rich foods, acrylamide forms through a chemical process called Maillard reaction involving reducing sugars and amino acids, such as asparagine (Fig. 1, Supplementary information). Presence of acrylamide gives the characteristic dark brown colour and texture of these food. Several studies have investigated the effect of acrylamide formation on human health, revealing potential risks associated with its consumption. Acrylamide has been classified as a probable human carcinogen by the International Agency for Research on Cancer (IARC) (IARC, 1994). Animal studies have demonstrated carcinogenic effects of acrylamide exposure, particularly concerning its association with tumours in various organs, including the mammary glands, thyroid, and adrenal glands. In addition to its carcinogenic potential, acrylamide is also known to exert neurotoxic effects. Studies in animals and humans have reported neurological symptoms and impairments associated with acrylamide exposure, including peripheral neuropathy, ataxia, and cognitive deficits. Epidemiological studies in humans have provided further evidence of potential health risks associated with dietary acrylamide intake. While findings are not always consistent, some studies have reported associations between dietary acrylamide intake and increased risks of certain cancers, including renal cell carcinoma, ovarian cancer, and endometrial cancer. The effect of acrylamide formation in processed foods on human health is a complex and multifaceted issue, which can be mitigated by using L-asparaginase.

L-asparaginase has garnered attention in the food industry for its ability to mitigate the formation of acrylamide in processed foods. By catalysing hydrolysis of amine group present on the sidechain of asparagine, L-asparaginase reduces the availability of this precursor molecule, thereby inhibiting acrylamide formation. Several studies demonstrated significant reductions in acrylamide levels in various food products, including potato chips, French fries, and bread, through the enzymatic treatment with L-asparaginase (Pandiselvam et al., 2024). L-asparaginase also hydrolyses glutamine at a significantly lower rate, hence It is important to find out some way to engineer the enzyme so that it can be more specific towards asparagine. Extensive site-directed mutagenesis studies led to development of mutant L-asparaginase with greater specificity and higher activity. These mutant enzymes are more beneficial for different applications.

### 1.3 Purification advancement and formulation improvement

Despite its immense potential, challenges persist in the purification and formulation of L-asparaginase for therapeutic and food applications. Here we delve into the critical role of streamlined purification methodologies in acquiring enzyme preparations of exceptional purity, crucial for their applicability in therapeutic and industrial domains, with specific attention devoted on distinguishing methods based on intracellular and extracellular proteins. Several methods of expression and purification of asparaginase has been developed over the years. The choice between extracellular and intracellular expression of L-asparaginase and subsequent purification depends on various factors, including target purity, yield requirements, scalability, and production costs. Extracellular enzyme purification requires simple methodology and offers better purity, but often shows reduced activity and total yield. Intracellular purification provides higher yields but requires more complex processing and may incur higher costs. Additionally, formulation strategies need to be optimized to ensure enzyme stability, efficacy, and compatibility with diverse food matrices. Liquid formulations offer convenience of preparation and immediate usability but may have shorter shelf life and require cold storage. Lyophilized formulations need extensive and challenging processing steps, but provide extended stability, easy storage and transportation options. Lyophilized enzymes require reconstitution and activation before use. Substrate specific enzyme formulation offers ease of application in the industry and will be of significant interest in future. Advancements in these areas are imperative to unlock the full potential of L-asparaginase in industrial application.

### 1.4 Application

The application of L-asparaginase enzyme in food processing represents a promising strategy for addressing food safety concerns, improving product quality, and extending shelf life. Scientific evidence supports its efficacy in reducing acrylamide formation, enhancing bread quality, extending shelf life in packaged food products, and potentially improving safety in meat and seafood products (Baskar et al., 2019). L-Asparaginase presents a promising solution for effectively reducing the formation of acrylamide (Jia et al., 2021). Additionally the enzyme have antimicrobial activity, which can be important for food preservation and packaging (Kamar et al., 2023). By examining purification techniques, formulation strategies, and future research directions, this review aims to contribute to a deeper understanding and draw some future research prospective of L-asparaginase enzyme formulations and its potential applications in unexplored diverse fields to mitigate health risk.

## 2 Mechanism of L-Asparaginase

L-asparaginase (EC 3.5.1.1) is a crucial enzyme widely known for its ability to catalyze the hydrolysis of L-asparagine into L-aspartic acid and ammonia. The detailed enzyme kinetics and catalytic mechanism was studied by several groups and molecular interactions has been extensively described by Lubkowski et al. (Lubkowski et al., 2020). Effective deamination of free and bound Asn by *E. coli* Asparaginase-II made it the first choice for applications in the food industry and cancer therapy (Fig. 1). Asparaginase also deaminates l-Glutamine but at a much slower rate. This side activity is partially responsible for adverse drug reaction in child acute lymphoblastic leukemia (ALL) therapy but may not be significant for food applications.

### 2.1 Acrylamide reduction mechanism

L-asparaginase plays a vital role in mitigating acrylamide formation by depleting the free asparagine available for the Maillard reaction. The mechanism involves the enzymatic hydrolysis of asparagine into aspartic acid and ammonia, both of which are non-reactive in acrylamide formation pathways. The stepwise process is as follows: i. Substrate Recognition and Binding: L-asparaginase specifically binds to L-asparagine at its active site, forming an enzyme-substrate complex. ii. Hydrolysis Reaction: The enzyme catalyzes the cleavage of the amide bond in asparagine, resulting in the formation of aspartic acid and ammonia. The absence of L-Asn in the food matrix prevents acrylamide formation via Maillard reaction during high-temperature cooking (Jia et al., 2021).

## 3 Improving substrate specificity of L-asparaginase

L-asparaginase enzyme plays a crucial role in acrylamide reduction in food during high temperature processing (Fig. 1). Additionally, L-asparaginase harbours glutaminase activity where it hydrolyses the side chain amine group of glutamine and converts it to glutamic acid. Glutamine is involved in providing energy, supporting the immune system, and serving as a precursor for the synthesis of other important molecules in the body. So, glutaminase activity of L-asparaginase enzyme may leads to shortage of glutamine requirement in human body. This depletion of glutamine may have various effects on normal cells, as they also rely on glutamine for various cellular processes (Nguyen et al., 2018). Loss of glutamate may lead to liver dysfunction as it plays an important role to maintain pH, homeostasis and ammonia detoxication through the intercellular glutamine cycle. Its depletion may affect the liver's ability to perform certain metabolic functions.

Several strategies have been used to reduce or abolish the side effects of L-asparaginase. Classical strategy involved screening of various naturally occurring strains of *Penicillium sizovae* 2DSST1 and *Fusarium proliferatum* DCFS10 found in environmental samples led to identification and isolation of L-asparaginase sans glutaminase activity (Doriya & Kumar, 2016; Freitas et al., 2021). Another solution involves using heterologous expression, where expressing the L-asparaginase enzyme in alternative host systems that naturally exhibit lower glutaminase activity. Selecting an organism that shows favourable high asparaginase specificity is a potential strategy to address this issue (Ashok et al., 2019). A more rational approach starts with detailed structure-function studies of L-asparaginase leading to identification of amino acids involved in substrate binding and catalysis. Site-directed mutagenesis helped in engineering several L-asparaginase variants that lacks glutaminase activity. These engineered enzymes are expressed in heterologous host like *E. coli* for large scale production (Tsegaye et al., 2024).

## 4 L-asparaginase purification techniques

Diverse purification strategies have been applied to purify L-asparaginase from different extracellular or intracellular sources. For intracellular L-asparaginase expression *Escherichia coli BL21 (DE3)* strain is the most preferable host but different expression vectors were used in different studies (Table 1A). *Erwinia carotovora, Pectobacterium carotovorum MTCC 1428* strains are well known for native intracellular L-asparaginase enzyme production with impressive enzyme activity of 1034 U/mg and 2020.91 U/mg respectively (Kumar et al., 2011; Warangkar & Khobragade, 2010). Highest unit activity of 5925.17 U/mg was obtained when *Pseudomonas aeruginosa* asparaginase gene was cloned into *E. coli* using pET-28a (+) vector (Saeed et al., 2018). A study obtained a thermostable L-asparaginase enzyme from *Thermococcus zilligii* strain which have an impressive unit activity of 5278 ± 32 U/mg using pET-22b(+) vector in *E. coli* (Zuo et al., 2015)*.* The affinity chromatography method was commonly used for intracellular L-asparaginase protein purification (Table 1A). We have expressed *E. coli* L-asp with two specific mutations (K288H/Y176F) that are known to increase protein stability and abolish glutaminase activity. This enzyme showed high activity of 233.8 U/mg and negligible glutaminase activity (unpublished data).

**Table 1A:** Intracellular L-asparaginase.

Source of the enzyme	expression vector	Purification technique description	Activity	Reference
*Dickeya chrysanthemi (DcL-ASNase)*	pET28a (+)	High Flow nickel affinity chromatography, Filter sterilization to remove endotoxins.	20,734.1 U/mg	(Saeed et al., 2022)
*Pyrococcus furiosus*	pET26b (+)	High-Select High Flow (HF) nickel affinity chromatography	11,203.5 U/mg	(Saeed et al., 2020)
*Bacillus amyloliquefaciens MKSE*	pET-28a (+)	Ni-sepharose 6 FF affinity chromatography	136.3 IU/mg	(Yim & Kim, 2019)
*Vibrio cholerae*	pMCSG7	crude extract was prepared by sonication, Ni^2+^ − NTA affinity chromatography and gel filtration chromatography.	648.9 U/mg	(Radha et al., 2018)
*Pseudomonas aeruginosa*	pET-28a (+)	Ni-NTA affinity chromatography	5925.17 U/mg	(Saeed et al., 2018)
*Pseudomonas fluorescens MTCC 8127*	pET-32a	Ni-NTA affinity chromatography	26 U/mg	(Sindhu & Manonmani, 2018)
*Paenibacillus barengoltzii* CAU904	pET28a^+^	Nickel-iminodiacetic acid (IDA) column chromatography	35.2 U/mg	(Shi et al., 2017)
*Saccharomyces cerevisiae*	pET15b-ASP1	Hi-Trap nickel-NTA affinity column chromatography and desalting.	196.2 U/mg	(Costa et al., 2016)
*Mesoflavibacter zeaxanthinifaciens*	pET-16b	Affinity purification using His-Bind kit (Novagen, USA)	687.1 units/mg	(Lee et al., 2016)
*E. coli K-12 strain(JM109) Double mutant*	pET28a (+)	Ni-NTA affinity chromatography and gel filtration column chromatography	233.8 U/mg	Jana et al., unpublished data

*For more detailed information, refer to the supplementary references.

For extracellular L-asparaginase production, very few researchers relied on making recombinant strain (Table 1B). Native L-asparaginase secreted out from *Streptomyces gulbargensis* has a unit activity of 2053 IU/mg (Amena et al., 2010). Although there was less complexity in secreted protein purification process, but the average activity was found 300–650 U/mg (Table 1B), which is very less in comparison to intercellular protein. For purification of the extracellular protein, a common line of action was taken; ammonium sulphate precipitation followed by dialysis, ion-exchange and gel-filtration chromatography (Table 1B).

**Table 1B:** Extracellular L-asparaginase.

Source of the enzyme	Purification technique description	Specific Activity	Reference
*Streptomyces rochei* subsp. *chromatogenes NEAE-K*	Culture filtration, ammonium sulfate followed by DEAE-Sepharose CL-6B ion exchange column	119.51 UI/mg	(El-Naggar & El-Shweihy, 2020)
*Bacillus subtilis MK072695*	ammonium sulfate (85 %) precipitation and dialysis using semi permeable membrane.	680.9 U/mg	(Ameen et al., 2020)
*Staphylococcus* sp. *MGM1*	ammonium sulfate fractionation, dialysis and ion-exchange chromatography.	1.63 IU/mg	(Ebrahimipour et al., 2020)
*Bacillus licheniformis*	ammonium sulfate (70 %) precipitation, dialysis, size exclusion chromatography.	36.08 U/mg	(Alrumman et al., 2019)
*Bacillus velezensis*	ammonium sulfate (70 %) precipitation, dialysis, size exclusion chromatography.	31.77 U/mg	(Mostafa et al., 2019)

*For more detailed information, refer to the supplementary references.

Extraction of intracellular proteins can be complex and costly, involving cell lysis and multi-step purification methods, which adds procedural intricacy and economic concerns. Extracellular protein expression offers direct access to proteins from culture media and purification with fewer steps. Despite being simpler and more cost-effective, extracellular protein purification may result in lower yield and lower catalytic activity compared to intracellular proteins. Both methods have distinct pros and cons, with the choice depending on specific research or industrial goals. For food processing applications, intracellularly expressed recombinant L-asparaginase with small affinity tag (like 6His-tag) can be used for large scale enzyme purification from *E. coli*.

## 5 Formulation of L-asparaginase

The effectiveness of L-asparaginase across these diverse applications hinges significantly on its formulation. The stability of L-asparaginase is crucial for its effective application, influenced by factors like temperature, pH, and the strength of the formulation matrix. These factors support the enzyme's shelf life and impact its stability. Formulation strategies, both for liquid and powder forms, involve selecting appropriate matrices based on their intended applications (Table 2). Stabilizers are added to mitigate challenges and ensure the enzyme retains its activity during storage and administration. In the formulation of L-asparaginase, preventing protein denaturation and aggregation is paramount, as these processes can compromise enzyme activity and efficacy. Strategies must prioritize maintaining the three-dimensional structure and minimizing aggregation for optimal enzymatic function.

**Table 2:** Enzyme formulation excipient.

Type of formulation	Excipient for the formulation	Description	Reference
Enzyme immobilization of L-asparaginase	alginate-gelatin‑calcium phosphate capsules	storage efficiency only reducing to 27 % at six months	(Shivakumar 2023)
Lyophilized formulation of pegylated L-asparaginase	Phosphate buffer, sucrose, L-arginine	A stable lyophilized slat free composition	(MENDIRATTA 2020)
Covalent Immobilization for fluid food model system	food-grade agarose (Agar) spheres and N-hydroxysuccinimide esters	storage stability and reusability with 93.21 and 72.25 % storage for 28 days	(Li et al., 2020)
Lyophilized formulation of Pegaspargase	dibasic sodium phosphate, monobasic sodium phosphate and sodium chloride, and sucrose	permitting a prolonged shelf life of at least 2 years when stored at 2–8 °C	(Faschinger & Sessler, 2019)
bioconjugate to improve pharmacokinetic	poly styrene-co-maleic acid (PSMA) nanoparticles	After 60 min of induction the native enzyme had just 50 % of its activity while PSMA still had about 70 % of their activity	(Varshosaz & Anvari, 2018)
Antileukemic agent ^Pr^KIDROLASE® (L-asparaginase)	Glycine and sodium hydroxide	Intramuscular or intravenous infusion drug	(SAS & Pharmatec, 2017)
thermostable liquid formulation	100 mM Glycine in the mixed buffer contained 40 mM sodium citrate buffer and 40 mM sodium phosphate buffer at pH 6.	Resulted in a dramatic increase in T_m_ of 4 °C, leading to high thermostability in response to the exposure of high temperature.	(Fu et al., 2015)
Conjugated formulation	L-asparaginase was conjugated with succinimidyl succinate derivative of polyethylene glycol (SS-PEG, MW 5000)	The specific activity as retained after PEG-ylation was 62.84 ± 8.2 %	(Vasudev et al., 2011)
Acrylaway^@^, Novozymes North America, Inc., USA	Glycerol, water, Sodium benzoate, Potassium sorbate	formula for use in food applications.	(Novozymes North America and USA, 2006)

### 5.1 Liquid formulation

The utilization and requirements of liquid forms of L-asparaginase, along with the careful selection of appropriate excipients for these formulations, play a crucial role in enhancing stability and efficacy. Liquid formulations present notable advantages, including simplified preparation, easy administration and improved bioavailability. However liquid formations are often unstable at room temperature and needs specialized storage conditions. The choice of excipients is crucial to ensure the stability, solubility, and bioavailability of the enzyme in liquid formulations. The suitable excipients for food application is glycerol, water, sodium benzoate, potassium sorbate (Novozymes North America 2006). Here glycerol plays a crucial role in enhancing the stability, solubility, and overall performance of enzymes in liquid formulations. The enzyme's thermostability experiences a noteworthy improvement when glycine is incorporated into a mixed buffer, combined with sodium citrate and sodium phosphate buffers, at a pH of 6 (Fu et al., 2015). Sodium benzoate and potassium sorbate works as a preservative in enzyme liquid formulations. It helps inhibit the growth of microorganisms, extending the shelf life of the formulation and ensuring the enzyme's stability over time. Sodium hydroxide acts as a pH modifier, helping to create an environment conducive to the stability and activity of the enzyme. Enzyme formulation for intramuscular or intravenous infusion drug contain glycine and sodium hydroxide as a preferred excipient (SAS & Pharmatec, 2017).

### 5.2 Lyophilized formulation

Lyophilization, also known as freeze-drying, is a widely used technique to increase the shelf life of proteins by removing water from the formulation. The excipient selection for lyophilize formulation needs to obtain some key features like i) Addition of cryoprotectants such as sugars, polyols, or amino acids helps protect the protein structure during freezing and drying processes. Most studies show that sucrose is the majorly used cryoprotectant for L-asparaginase enzyme's lyophilize formulation as it helps to reduce the risk of protein denaturation and aggregation (Faschinger & Sessler, 2019, (Mendiratta, Patel, Patel, Bhatt and Chandresh, 2020)). ii) A suitable buffer system like phosphate buffer (combination of dibasic sodium phosphate, monobasic sodium phosphate) is essential to maintain the L-asparaginase enzyme's stability and prevent pH-induced degradation (Faschinger & Sessler, 2019, MENDIRATTA 2020). Buffers can also play a role in controlling ice crystal formation during freezing. iii) Sodium chloride acts as an osmotic stabilizer during the freezing and drying stages of lyophilization (Faschinger & Sessler, 2019). By controlling the osmotic pressure, NaCl minimizes the potential for disruption of protein structure and maintains the integrity of the protein during the process. iv) In some cases inert fillers or bulking agent like mannitol may be added to the formulation to improve the physical properties of the lyophilized power (Thakral et al., 2023).

Lyophilization can significantly extend the shelf life of the enzyme by removing water, which is a major factor in degradation reactions (Putranto & Chen, 2019). Removal of water reduces the weight and volume of the product, resulting in lower shipping and storage costs. On the other hand, there is some limitation of lyophilization. It is a complex process that requires precise control of freezing, primary drying, and secondary drying steps. Variations in these conditions can impact the quality of the lyophilized product (Juckers et al., 2023).

### 5.3 Other formulation technique

Enzyme formulation techniques go beyond liquid and lyophilization formulations, the immobilization and conjugation are two additional strategies employed for L-asparaginase enzyme applications. i) Immobilization refers to the process of fixing enzymes onto a solid support or matrix, which can be natural or synthetic. One study showed that L-asparaginase enzyme immobilized in alginate-gelatin‑calcium phosphate capsules lead to storage efficiency reducing to 27 % at six months storage time (Shivakumar and a. S., 2023). Another study conclude that covalent immobilization of L-asparaginase for fluid food model with food-grade agarose (Agar) and N-hydroxy succinimide esters can enhance storage stability and reusability with 93.21 % and 72.25 % storage for 28 days (Li et al., 2020). ii) Conjugation involves attaching other molecules or ligands to enzymes to modify their properties or confer additional functionalities. The polyethylene glycol (PEG) is commonly used molecule for conjugation with L-asparaginase enzyme. During encapsulation, a significant portion of the native protein becomes denatured, leading to the formation of insoluble aggregates. To address this issue, L-asparaginase was modified through PEGylation prior to exposure to harsh conditions. This modification improved the stability of the protein in the presence of the organic solvent. However, it was reported that approximately 62.84 ± 8.2 % of the specific activity was retained following PEGylation, contributing to enhanced stability. Bioconjugation with poly styrene-*co*-maleic acid (PSMA) nanoparticles helps in retention 70 % activity after 60 min of while the native enzyme can retain only 50 % of its activity (Varshosaz & Anvari, 2018). Each formulation technique has its advantages and is chosen based on the specific requirements of the application. The choice depends on factors such as the desired enzyme properties, the intended use, and the environmental conditions of the process.

Some advanced formulation technique was reported where enzymes are encapsulated within microspheres or beads, providing protection and controlled release. Inclusion Complexes, where the enzymes are incorporated into host molecules, Virus-like Particles (e.g., Brome Mosaic Virus), often enhances enzyme stability (Villanueva-Flores et al., 2023).

## 6 Healthcare applications of L-asparaginase and future aspects

The healthcare applications of L-asparaginase enzyme are primarily centred around its role in cancer treatment, particularly in the context of haematological malignancies such as acute lymphoblastic leukemia (ALL). L-asparaginase is employed as a therapeutic agent due to its ability to deplete circulating asparagine, an essential amino acid for the growth and survival of cancer cells. L-asparaginase catalyzes the hydrolysis of asparagine into aspartic acid and ammonia. Many cancer cells, especially leukemic cells, lack the enzyme asparagine synthetase and are unable to synthesize their own asparagine. Thus, they depend on external sources of asparagine for protein synthesis and cell proliferation. L-asparaginase creates an asparagine-deficient environment, leading to impaired protein synthesis and ultimately inducing apoptosis (programmed cell death) in cancer cells.

Combination Therapies: Biotechnological advances allow for the exploration of combination therapies, where L-asparaginase is used in conjunction with other chemotherapeutic agents to enhance the overall efficacy of cancer treatment (Al-Dulimi et al., 2020).

Future research on the healthcare application of L-asparaginase enzyme could explore several avenues to improve its efficacy, reduce side effects, and broaden its therapeutic applications. Some potential research approaches are nanoparticle -based drug delivery systems to enhance the targeted delivery of L-asparaginase to cancer cells, (Chehelgerdi, Chehelgerdi et al., 2023). synergistic combinations of L-asparaginase with other chemotherapeutic agents or targeted therapies and study the immunomodulatory effects of L-asparaginase (Möricke et al., 2023).

## 7 Application of L-asparaginase in food industry

The International Agency for Research on Cancer (IARC) classifies acrylamide as a probable human carcinogen, linked to cancer in organs like the kidneys, ovaries, and uterus, with potential neurotoxic effects. The application of L-asparaginase in the food industry offers a promising approach to mitigate the formation of acrylamide, thereby reducing potential health risks associated with its consumption. By selectively targeting the precursor molecule (asparagine), L-asparaginase effectively disrupts the Maillard reaction pathway, leading to decreased acrylamide formation without compromising the sensory attributes of the final product (Jia et al., 2021). However, it is essential to consider several factors when implementing L-asparaginase in food processing. These include enzyme stability, optimal conditions for activity, potential impact on food flavour and texture, and regulatory approval for its use in food applications. Additionally, in food processing, L-asparaginase can be applied in various ways; i) Pre-treatment: foods rich in asparagine, such as potatoes, can be treated with L-asparaginase before cooking. This helps to reduce the substrate available for acrylamide formation during subsequent cooking processes. ii) Incorporation into formulations: L-asparaginase can be added directly to food improver formulations to enzymatically degrade asparagine present in the ingredients. This is particularly useful in the production of baked goods, snacks, and fried products.

### 7.1 Acrylamide reduction profile in different food matrix

Acrylamide is commonly formed in three types of processed food matrix a) fried food, b) baked food and c) some beverages. L-asparaginase from different source represents a valuable enzymatic tool for addressing the issue of acrylamide formation in the food. Its application offers a proactive approach to food safety and public health by minimizing the presence of this potentially carcinogenic compound in processed foods. The majorly worldwide consumed fried foods are french fries, potato chips and doughnuts, which have high acrylamide content. Previous studies shows that the acrylamide reduction was reported in French fries, potato chips & doughnuts respectively up-to 80 %, 80–94 % & up-to 90 % (Table 3A). The thermostable l-asparaginase enzyme obtained from *Thermococcus zilligii AN1 TziAN1_1* has the maximum acrylamide reduction capacity of 80.5 % (Zuo et al., 2015). Enzyme extract from *Fusarium culmorum (ASP-87)* was applied on fried potato chips and shows the maximum acrylamide reduction of 94 % (Meghavarnam & Janakiraman, 2018). One study shows up-to 90 % acrylamide reduction in fried-dough pastry processing by treating with the commercially available L-asparaginase enzyme (Novozymes, Denmark) (Kukurová et al., 2009).

**Table 3A:** Acrylamide reduction table in fried food.

Source of the enzyme	Food matrix	Acrylamide reduction	Reference
*Rhizomucormiehei, Bacillus subtilis, Thermococcus zilligii*	Potato chips and French fries	**80 %**	(Sundarrajan et al., 2022)
*Fusarium culmorum (ASP-87)*	fried potato chips	**94 %**	(Meghavarnam & Janakiraman, 2018)
*Paenibacillus barengoltzii* CAU904	potato chips	**86 %**	(Shi et al., 2017)
*Pseudomonas oryzihabitans*	Potato chips	**90 %**	(Bhagat et al., 2016)
*Candida utilis*	Potato Crisps	**58 %**	(Torang & Alemzadeh, 2016)

*For more detailed information, refer to the supplementary references.

In case of baked food, enzyme extract from *Fusarium culmorum (ASP-87)* has the max acrylamide reduction profile of 86 % (Meghavarnam & Janakiraman, 2018). The commonly uptake baked food like sweet bread, wood oven baked pizza bases, Biscuits & mooncakes which have the high acrylamide content; after treating with L-asparaginase enzyme the acrylamide level reduced 81 %, 50–60 %, 84 % and 52 % respectively (Table 3B).

**Table 3B:** Acrylamide reduction table in backed food.

Source of the enzyme	Food matrix	Acrylamide reduction	Reference
*Aspergillus niger* (Preventase®W, Preventase®M, Preventase®XR-BG)	wood oven baked pizza bases	**50–60 %**	(Covino et al., 2023)
*Fusarium culmorum (ASP-87)*	baked bread	**86 %**	(Meghavarnam & Janakiraman, 2018)
*Paenibacillus barengoltzii* CAU904	mooncakes	**52 %**	(Shi et al., 2017)

*For more detailed information, refer to the supplementary references.

**Fig. 2:**
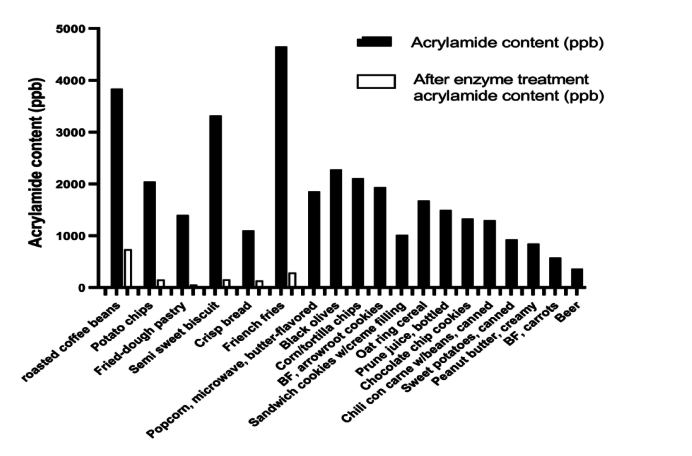
**Acrylamide reduction profile of different food matrices before and after L-asparaginase treatment.** Acrylamide content (ppb) in commonly studied food matrices is shown as filled bar. White boxes represent the level of acrylamide in the same matrix after L-asparaginase treatment. The values were taken from available literature and listed in Table 3A-3B.

The popular hot beverage like coffee have high acrylamide content, it was reported that the green coffee beans contain 200–1000 mg/kg of acrylamide. Upon enzyme treatment of light and dark roasted coffee beans, the acrylamide content reduced up to 80.7 % & 75.8 % (Khalil et al., 2021) (Table 3C). One research group applied commercially available enzyme Acrylaway™ (Bagsvaerd, Denmark) during coffee brewing process and observed 77 % reduction of acrylamide content (Corrêa et al., 2021). According to U.S. FDA there are several other unexplored processed foods along with well-studied food matrices available in the market that have high acrylamide content (Fig. 2). L-asparaginase from different sources exhibit significant potential in reducing acrylamide content across various food matrices, offering promising solutions to mitigate the potential health risks associated with acrylamide consumption.

**Table 3C:** Acrylamide reduction table in beverage.

Source of the enzyme	Food matrix	Acrylamide reduction	Reference
Asparaginase solution (Acrylaway™, Bagsvaerd, Denmark)	coffee	**77 %**	(Corrêa et al., 2021)
*Penicillium crustosum* L-asparaginase	roasted coffee beans	**80.7 %** and **75.8 %** for light and dark roasted beans, respectively.	(Khalil et al., 2021)

### 7.2 Alternative methods for prevent acrylamide formation

Preventing acrylamide formation in fried and bakery foods involves a multifaceted approach. The catalytic action of L-asparaginase enzyme provides a targeted enzymatic solution, while using alternative strategies with the enzyme can leads to better result, such as adjusting pH, controlling frying temperatures, employing blanching techniques, and managing sugar content in bread as effective means to reduce acrylamide levels significantly. A comprehensive understanding of these methods will contribute to the development of healthier food products with lower acrylamide content, addressing both consumer concerns and regulatory standards.

i. Adjusting pH to lower acrylamide formation: Research has shown that the pH of the food matrix plays a crucial role in acrylamide formation. Lowering the pH can reduce the concentration of acrylamide precursors and disrupt the Maillard reaction. This method, however, requires careful optimization to avoid affecting the sensory properties of the food product. In a study by Kalita and Jayanty (2013), treating potato slices with a 0.1 M vanadyl oxysulfate (VOSO₄) solution resulted in a substantial 92.5 % reduction in acrylamide formation (Kalita & Jayanty, 2013). However, currently the cost associated with this treatment is approximately $76.6 per kg of potatoes, which may limit its economic feasibility for large-scale food processing applications. The vanadium ions, particularly VO^2+^, are known to interact with amino acids such as asparagine, forming complexes. The binding of VO^2+^ to asparagine may disrupt or alter the reactivity of asparagine in the Maillard reaction, potentially leading to a reduction in acrylamide formation. Lowering the pH can alter the reaction pathways and reduce the likelihood of acrylamide formation (Mestdagh et al., 2008). Simultaneously decreasing the pH of the potato samples may further contribute to this reduction.
ii. Frying temperature control: The frying temperature significantly influences acrylamide formation. Higher temperatures with cooking time are associated with increased acrylamide content in fried foods. Implementing controlled frying temperatures within optimal ranges can effectively lower acrylamide levels while ensuring the desired sensory attributes of the food product. It was reported that reducing the frying temperature from 180 °C to 165 °C and further to 150 °C resulted in a 6 % and 18 % decrease in acrylamide content after 4 min, respectively (Torang & Alemzadeh, 2016).
iii. Blanching as a pre-treatment method: Blanching, a pre-treatment involving the brief exposure of food to high temperatures, has been explored to reduce acrylamide formation. By partially boiling the food before the final processing steps, blanching can decrease the concentration of starch from the food matrix, providing an additional layer of control over the formation of this carcinogenic acrylamide compound. In a study, potato chips produced 2047 mg/kg of acrylamide when not subjected to blanching. However, incorporating the blanching process led to a nearly 17 % reduction, with only 1697 mg/kg of acrylamide formed in potato slices after frying (Pedreschi et al., 2011).
iv. Managing sugar content in bread: The sugar content in bread has been identified as a contributing factor to acrylamide formation during baking. Reducing the sugar content in bread formulations or exploring alternative sweeteners may offer a practical approach to minimize acrylamide levels in bakery products. The substitution of ammonium hydrogen carbonate with sodium hydrogen carbonate as a baking agent and the replacement of traditional sugar syrup with sucrose in the wheat cracker preparation led to a remarkable reduction of almost 80 % in acrylamide content (Vass et al., 2004).

There are some other techniques like CaCl_2_ and NaCl salt treatment of the food matrix. In french fries preparation, frozen pre-fried products are sometimes used to simplify the process. However, studies indicate that enzyme-applied, blanched, chilled French fries surpass the quality of frozen pre-fried alternatives. This improvement is attributed to the elongated enzyme-substrate reaction time during the process, resulting in more effective reduction of acrylamide (Medeiros Vinci et al., 2011).

## 8 Industrial implementation strategy and challenges

The industrial application of L-asparaginase in food processing to reduce acrylamide levels offers a significant opportunity to address global public health concerns related to dietary exposure to this carcinogenic compound. However, scaling up the use of this enzyme presents both technical and economic challenges. By examining how L-asparaginase compares to other acrylamide mitigation strategies, we highlight its potential to serve as an effective, industry-wide solution to enhance food safety and protect public health.

### 8.1 Economic feasibility of large-scale implementation

The economic feasibility of incorporating L-asparaginase into food processing is influenced by factors such as production costs, enzyme stability, and scalability. While initial investments in enzyme production and the adaptation of existing manufacturing processes pose challenges, the long-term benefits, particularly compliance with acrylamide reduction regulations established by authorities like the European Food Safety Authority (EFSA), justify the expenditure. This is especially relevant for high-acrylamide containing foods like potato chips and baked goods.

In commercial applications, the enzyme dosage is calibrated based on the asparagine content within the food matrix. According to a report by Novozymes (Hendriksen et al., 2009), optimal dosages of the commercial enzyme Acrylaway® have been identified for various food products: potato chips (1000 ASNU/L), french fries (10,500 ASNU/L), semisweet biscuits (525 ASNU/kg), crispbread (2100 ASNU/kg), and ginger biscuits (1000 ASNU/kg). An economic analysis (Table 4) suggests that the cost-effectiveness of L-asparaginase treatment is viable at an industrial scale. For instance, treating french fries with the optimal enzyme dosage incurs an enzyme cost of approximately US$ 0.324 per kg of potatoes. For example, a standard portion of McCain french fries (750 g) without enzyme treatment currently costs approximately US$ 17.10, whereas the cost for enzyme-treated, acrylamide-free french fries will be around $17.34. This marginal increase (∼4 %) in cost will be negligible for the food industry, especially when weighed against the public health benefits associated with reduced acrylamide exposure.

**Table 4:** Economic analyses L-asparaginase commercial product (Acrylaway®, PreventASe L®) according to there optimal dosages.

Food Matrix	L-ASNase	Enzyme Dosage with References	Acrylamide Reduction	Cost (per kg food matrix)
Homemade bread	Acrylaway 3500 BG® (granulated)	300 U/kg flour (Calabrese et al., 2024)	78 %	∼0.0095 USD
French fries	PreventASe L®	25,000 ASPU/L (Rottmann et al., 2021)	59 %	0.5 USD
French fries	Acrylaway® (granulated)	10,000 ASNU/L (Pedreschi et al., 2008)	60 %	∼0.308 USD
French fries	AcrylawayL® (liquid)	10,500 ASNU/L (Hendriksen et al., 2009)	59 %	∼0.324 USD
Potato chips	AcrylawayL® (liquid)	10,000 ASNU/L (Hendriksen et al., 2009)	62 %	∼0.308 USD
Semisweet Biscuits	AcrylawayL® (liquid)	525 ASNU/kg of flour (Hendriksen et al., 2009)	65 %	∼0.016 USD
Crisp Bread	AcrylawayL® (liquid)	2100 ASNU/kg of flour (Hendriksen et al., 2009)	84–92 %	∼0.064 USD
Ginger Biscuits	AcrylawayL® (liquid)	1000 ASNU/kg of flour (Hendriksen et al., 2009)	34–90 %	∼0.03 USD
Turkish coffee	AcrylawayL® (liquid)	2126.4 ASNU/kg green bean (Akgün et al., 2021)	Not reported	∼0.065 USD
Arabica coffee	Acrylaway™ (granulated)	5000 ASNU/kg (Corrêa et al., 2021)	59 %	∼0.154 USD

*Cost per kg food matrix was calculated using the 2024 price of the commercially available enzyme in market.

Recent studies by Calabrese et al. (2024) demonstrate that utilizing an enzyme variant such as Acrylaway 3500 BG® at 300 U/kg of flour in homemade bread can decrease acrylamide levels by up to 78 % (Calabrese et al., 2024), potentially leading to reduced production costs. Additionally, Rottmann et al. (2021) reported that using PreventASe L® for treating french fries results in an additional cost of $0.50 per kg (Rottmann et al., 2021).

To summarise the additional cost associated with acrylamide mitigation varies across different food matrices, the added cost ranges from $0.3 to $0.5 per kg for potato-based fried products, $0.01 to $0.06 per kg for flour-based baked goods, and $0.07 to $0.15 per kg for coffee bean-based beverages. These costs are deemed economically feasible, particularly in light of the significant health advantages gained from reduced acrylamide consumption.

### 8.2 Toxicity concerns

Ensuring safety is of utmost importance when introducing enzymes into the food supply chain. L-asparaginase derived from bacterial sources such as *Escherichia coli* and fungal sources like *Aspergillus oryzae* is generally recognized as safe (GRAS) by the U.S. Food and Drug Administration (FDA). Notably, ELSPAR®, an FDA-approved injectable form of L-asparaginase derived from *E. coli*, is widely used in the treatment of acute lymphoblastic leukemia, demonstrating a well-established safety profile in clinical applications. This supports its potential safe use in food applications.

However, concerns remain regarding potential allergenicity and the presence of residual enzyme activity in the final food product. A recent toxicological evaluation conducted by the European Food Safety Authority (EFSA) Panel on Food Contact Materials (2023)(EFSA Panel on Food Contact Materials, E, 2023) concluded that residual L-asparaginase in food products did not exhibit cytotoxic or genotoxic effects in vitro under the intended conditions of use (EFSA Panel on Food Contact Materials, E, 2023), Aids et al. 2023). These findings support the enzyme's safety for consumption within regulated limits.

### 8.3 Efficiency and cost-effectiveness of different sources

Microbial sources like *Escherichia coli* and *Aspergillus niger* produce high yields of L-asparaginase, potentially lowering production costs compared to traditional chemical acrylamide reduction methods. Fungal L-asparaginase from *Aspergillus* strains shows excellent stability across a pH range of 5–8, enhancing its suitability for various food matrices (Dias et al., 2019). However, no data exist on the production cost of L-asparaginase in yeast, preventing direct comparisons with bacterial and fungal enzymes. Utilizing engineered yeast that secretes L-asparaginase during baking could offer a cost-effective approach for acrylamide mitigation in bread, but this approach may be limited by restriction of use of genetically modified organisms (GMO) for food.

Notable high-activity enzyme producers include *Dickeya dadantii* (20,734.1 U/mg), *Pseudomonas aeruginosa* (5925.17 U/mg), and *Pyrococcus furiosus* (11,203.5 U/mg). These organisms do not fall under GRAS, hence can't be used directly for food application. If the recombinant enzymes expressed in *E. coli* show comparable activity and conform safety and regulatory guidelines, then they may be tested for food applications. Taken together, there are diverse possibilities for application of asparaginase in food processing in a low-cost and economically viable manner even at a large scale.

### 8.4 Impact on taste and texture

The enzymatic treatment must not alter the sensory attributes of food. L-asparaginase has been shown to have minimal impact on taste and texture when applied under controlled conditions. A sensory evaluation study conducted by Rottmann et al. demonstrated that L-asparaginase-treated french fries were indistinguishable from untreated controls in terms of taste, crispness, and colour, while achieving a 59 % reduction in acrylamide levels (Rottmann et al., 2021).

Nonetheless, the enzyme's interaction with certain contents, such as proteins and fats, could potentially affect flavour profiles in complex matrices like baked goods. Optimization of enzyme dosage and processing time is critical to minimizing any adverse effects on sensory quality.

### 8.5 Stability of L-asparaginase in food matrices

The stability of L-asparaginase is crucial for its effectiveness, particularly in packaged foods with extended shelf lives. Enzyme application in food processing is typically a short-duration step, and its effectiveness varies based on the food matrix. For instance, in the processing of french fries, excess enzyme solution is removed after incubation, whereas in dough-based baked products, the enzyme remains integrated within the food matrix.

Subsequent high-temperature processing, such as baking or frying, will lead to denaturation and possible degradation of any residual enzyme, thereby minimizing its presence in the final product. This degradation ensures that the enzyme does not remain active in the food, which is particularly relevant for maintaining the safety and quality of the treated food products.

## 9 Future research area

Researchers are actively working on optimizing L-asparaginase production to get high protein yield with substantial enzymatic activity. It is reported that the mutant *Erwinia chrysanthemi* L-asparaginase has a very low glutaminase activity. As secreted protein purification process is simpler and more cost-effective, a genetically engineered glutaminase free and more active extracellular L-asparaginase enzyme production for food industrial application may be attempted. Our laboratory has produced a modified *E. coli* L-asparaginase with negligible glutaminase activity and high yield in *E. coli*. This enzyme can be tested for different applications.

While there is substantial research on acrylamide reduction in commonly consumed items, there is a lack of comprehensive studies explaining how acrylamide content can be minimized in different food matrices that may pose health risks. The Council for Agricultural Science and Technology (CAST) established a No Significant Risk Level (NSRL) for acrylamide in 1990, initially set at 0.2 g acrylamide/person/day (David Lineback et al., 2006). This level was subsequently modified in 2005, with an alternative NSRL of 1.0 g acrylamide/person/day established for bread and breakfast cereals (David Lineback et al., 2006).

**Fig. 3:**
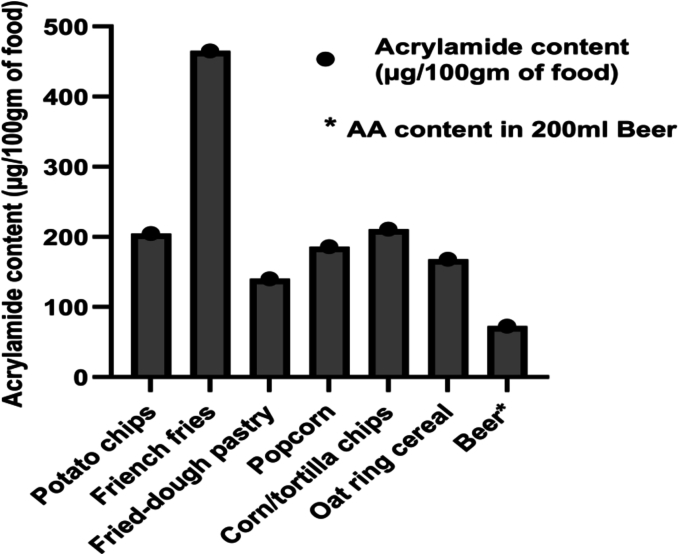
**Acrylamide content in popular food products.** Acrylamide content in other popular food products was calculated as amount of acrylamide (μg) present per 100 g food (as reported in the literature). For beer it is amount of acrylamide (μg) per 200 ml liquid.

It is noteworthy that certain popular food items, including popcorn, black olives, corn/tortilla chips, various cookies, oat ring cereal, prune juice, sweet potatoes, peanut butter, carrots, and beer exceed the minimum risk level (Fig. 3) for acrylamide content. A study by Hasan et al. (2021) showed that the average acrylamide contents in grilled chicken, beef grill, grilled fish were 80.27 μg/Kg, 81.52 μg/Kg and 48.39 μg/Kg respectively (Hasan et al., 2021). To ensure better health outcomes, attention should be directed towards mitigating acrylamide levels in these food matrices that surpass the NSRL. Preliminary studies suggest the feasibility of L-asparaginase treatment in meat and seafood processing.

L-Asparaginase exhibits antimicrobial properties that can inhibit the growth of spoilage microorganisms in packet food products like raw fish fillet and meat. Addition of L-asparaginase to the food formulations has been shown to extend shelf life by suppressing bacterial growth and delaying product deterioration. Kumar et al. (2023) illustrated the antimicrobial activity of L-asparaginase against *Listeria monocytogenes* (Kamar et al., 2023)*.*

For enzyme application it is also important to prepare proper formulation of enzyme according to the food matrix. In the modern bakery industry, bread improvers are widely utilized and recognized for their role in enhancing the quality of bread. Future research could be directed towards integrating L-asparaginase enzyme directly into bread improvers. This innovation would enable the enzyme-improver to serve a dual function within the bakery industry, offering both quality enhancement and acrylamide reduction benefits. Such a product would not only streamline production processes but also enhance user-friendliness, representing a significant advancement in bakery ingredient technology.

Advancements in enzyme engineering helped in development of more efficient, temperature tolerant and cost-effective L-asparaginase enzymes. Development of different easy to use enzyme formulation can lead to easy incorporation in food processing systems to reduce acrylamide formation without compromising taste, texture, or appearance of the final product. The demand for low-acrylamide or acrylamide-free food products is creating new market opportunities for companies that can offer innovative solutions. As concerns about acrylamide in food continue to grow, the demand for L-asparaginase enzyme as a solution to mitigate its formation is likely to increase in the food application industry.

## 10 Conclusion

The rising awareness of the health risks associated with acrylamide consumption has sparked a significant demand for low-acrylamide or acrylamide-free food products, highlighting the crucial role of L-asparaginase in the food industry. In 2016, the U.S. FDA issued a Guidance for Industry on Acrylamide in Foods, recognizing acrylamide as a potential health concern. However, no specific maximum allowable level of acrylamide in food has been established to date. Earlier, a comprehensive evaluation by the World Health Organization (WHO) and the Food and Agriculture Organization (FAO) of the United Nations through the Joint Expert Committee on Food Additives (JECFA) in 2010 concluded that acrylamide could be “a human health concern” (Nations, 2011). L-asparaginase offers a cost-effective solution for acrylamide mitigation, adding only an about US$ 0.3–0.5 per kg to the food matrix. It can be easily incorporated in the existing food processing methods thus will need minimal process optimization. Acrylamide treatment will minimally affect the organoleptic properties of the product, thus mitigating the risk of low acceptability of the product.

L-asparaginase exhibits remarkable stability over prolonged periods when combined with stabilizing agents such as alginate-gelatin‑calcium phosphate capsules, sucrose, and L-arginine, suitable for both liquid and lyophilized formulations. Beyond acrylamide mitigation, L-asparaginase also possesses inherent antimicrobial properties, which can extend the shelf life of food products by inhibiting spoilage microorganisms. This dual functionality makes L-asparaginase an asset in modern food processing, offering potential for diverse formulations that ensure comprehensive food preservation.

Extensive research has demonstrated the efficacy of L-asparaginase in reducing acrylamide levels in commonly studied food matrices like French fries, potato chips, bread, cookies, and coffee. However, there remain several popular food matrices, such as popcorn, corn/tortilla chips, and beer; that are known to contain significant acrylamide levels but have not yet been explored extensively. Future studies should focus on these under-researched areas to fully leverage the enzyme's potential in food safety and quality enhancement.

## CRediT authorship contribution statement

**Arindam Jana:** Writing – review & editing, Writing – original draft, Data curation, Conceptualization. **Soumyajit Biswas:** Writing – original draft, Data curation. **Ritu Ghosh:** Writing – original draft. **Rahul Modak:** Writing – review & editing, Supervision, Project administration, Data curation, Conceptualization.

## Declaration of competing interest

The authors declare that they have no known competing financial interests or personal relationships that could have appeared to influence the work reported in this paper.

## Data Availability

No data was used for the research described in the article.

## Appendix A Supplementary data

Supplementary material: Mechanism of Maillard Reaction and Supplementary tables Image 1
