# Supplementary material for: Recent advances in L-Asparaginase enzyme production and formulation development for acrylamide reduction during food processing
Source: Food Chem X. 2024 Dec 2;25:102055. doi: 10.1016/j.fochx.2024.102055 (PMC11696629; doi:10.1016/j.fochx.2024.102055)
Supplement: Supplementary file 1 — Supplementary material: Mechanism of Maillard Reaction and Supplementary tables [file mmc1.docx]

**Recent advances in L-Asparaginase enzyme production and formulation development for acrylamide reduction during food processing**

**Authors:**

Arindam Jana^1,2^, Soumyajit Biswas^1^, Ritu Ghosh^1,3^ and Rahul Modak^1,^*

**Affiliations:**

1. Infection and Epigenetics Group, School of Biotechnology, Kalinga Institute of Industrial Technology, Bhubaneswar: 751024, Odisha, INDIA
2. KIIT - Technology Business Incubator (KIIT-TBI), KIIT-DU, Bhubaneswar: 751024, Odisha, INDIA
3. University of Tartu: Faculty of Science and Technology, Institute of Technology, Nooruse 1, 50411 Tartu, ESTONIA

* To whom correspondence should be addressed. Tel: +91-9040442305, +91-674- 2725466,

Email: [rahul.modak@kiitbiotech.ac.in](mailto:Rahul.modak@kiitbiotech.ac.in), [rahulmodak123@gmail.com](mailto:rahulmodak123@gmail.com)

**Maillard reaction mechanism:**

The Maillard reaction is a complex chemical process responsible for the browning and flavor development in cooked foods, occurring in three distinct stages. The early stage begins at high temperatures when reducing sugars react with amino groups of proteins, peptides, or free amino acids. This initial interaction forms an unstable Schiff base through a nucleophilic addition reaction. The Schiff base undergoes tautomerization to produce an enaminol intermediate, which then rearranges into a more stable ketoamine structure, commonly referred to as the Amadori product (or Heyns product in the case of ketose sugars). This stage sets the foundation for the subsequent reactions by creating reactive intermediates.

During the intermediate stage, the Amadori product undergoes further transformations, including enolization, dehydration, and fragmentation, leading to the formation of highly reactive dicarbonyl compounds such as glyoxal, methylglyoxal, and diacetyl. These intermediates are pivotal as they act as precursors for the advanced reactions, significantly contributing to the complexity of the Maillard pathway.

In the final stage, these reactive intermediates interact with additional amino acids, sugars, or other compounds, forming a diverse array of products, including advanced glycation end-products (AGEs) and melanoidins. AGEs are implicated in altering the texture and bioactivity of the food, while melanoidins, the polymeric brown pigments, impart characteristic changes in the aroma, flavor, and color of the final product. This stage is responsible for the rich sensory qualities associated with baked, roasted, or fried foods, though it may also have implications for food safety and health due to the formation of potentially harmful AGEs under certain conditions. The Maillard reaction exemplifies the intricate interplay of chemical reactions that transform simple food components into complex sensory experiences (Rauh and Xiao 2022).

**Supplementary figure 1:** Acrylamide formation in food through Maillard reaction.


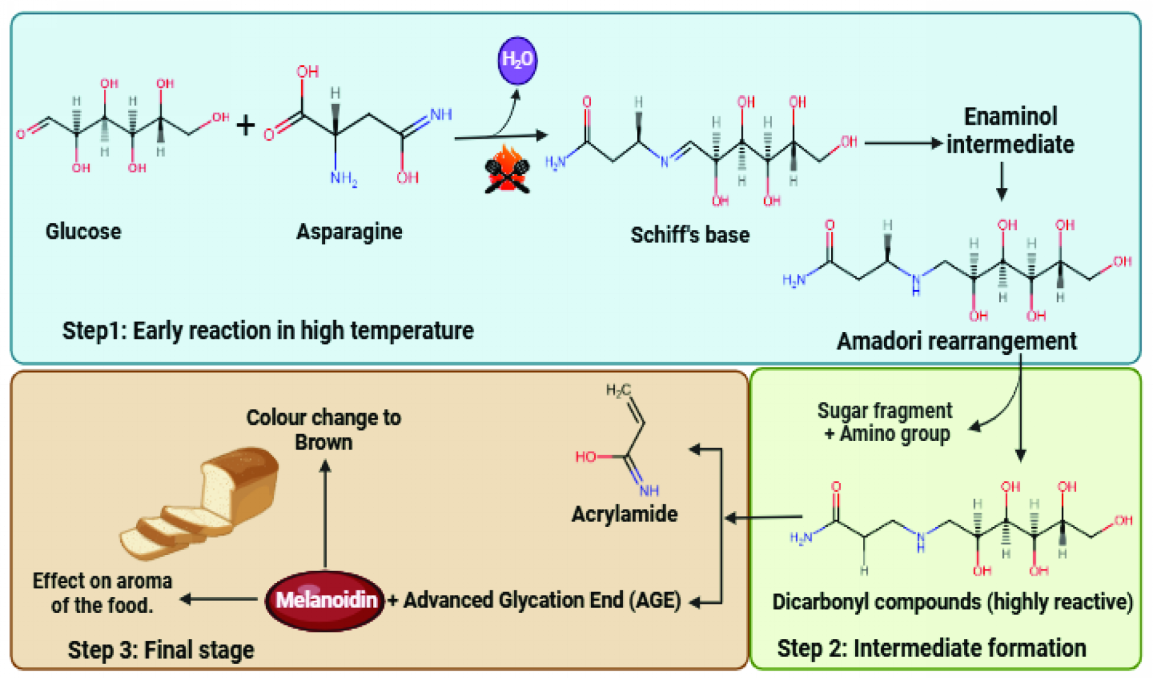


**Table 1A: Intracellular L-asparaginase**

| **Source of the enzyme** | **expression vector** | **Purification technique description** | **Activity** | **Reference** |
| --- | --- | --- | --- | --- |
| thermostable l-asparaginase from *Thermococcus zilligii AN1 TziAN1_1* | pET-22b(+) | Nickel affinity chromatography, dialyzed twice against 50 mM Tris–HCl buffer (pH 8.5) and gel filtration column chromatography | 5278 ± 32 U/mg | (Zuo, Zhang et al. 2015) |
| *E. coli* K-12 strain(JM109) | pET14b | Isolated asparaginase IBs, pulsatile dilution method, dialysis by buffer exchange, Refolded L-asparaginase was purified using DEAE-sepharose anion exchange chromatography and S-200 Sephacryl gel-filtration chromatography. | 190 IU/mg | (Upadhyay, Singh et al. 2014) |
| *Pectobacterium carotovorum MTCC 1428* | **-** | Ammonium sulfate precipitation, DEAE cellulose ion-exchange chromatography and Sephadex G-100 gel filtration chromatography | 2020.91 IU/mg | (Kumar, Dasu et al. 2011) |
| *Yersinia pseudotuberculosis Q66CJ2 (YpA)* | pBad24 | Sonication extraction, two-stage anion exchange chromatography using Q-Sepharose (1.5×16.0 cm) & DEAE-Toyopearl (1.0×12.5 cm) column | 62.7 U/mg | (Sidoruk, Pokrovsky et al. 2011) |
| *Erwinia carotovora* | **-** | ammonium sulfate fractionation (60–70%), Sephadex G-100 gel filtration, DEAE sephadex ion exchange chromatography | 1034 IU/mg | (Warangkar and Khobragade 2010) |

**Table 1B: Extracellular L-asparaginase**

| **Source of the enzyme** | **expression vector** | **Purification technique description** | **Specific Activity** | **Reference** |
| --- | --- | --- | --- | --- |
| *Streptomyces brollosae NEAE-115* | *-* | ammonium sulphate precipitation (55–85%) and pure enzyme was reached using ion-exchange chromatography | 76.671 U/mg | (El-Naggar, Deraz et al. 2018) |
| *Aspergillus niger* AKV-MKBU | *-* | Ultra-filtration, ethanol precipitation and ion-exchange chromatography. | 46.75 U/mg | (Vala, Sachaniya et al. 2018) |
| *Aspergillus oryzae CCT 3940* | *-* | Crude extract, Ammonium sulfate precipitation, Ion-exchange chromatography | 282 U/mg | (Dias, Ruiz et al. 2016) |
| *Erwinia chrysanthemi NCPPB1125* | pUC57 (pUaspg) | Gel filtration and ion-exchange chromatography. | 312.8 U/mg | (Nguyen, Nguyen et al. 2016) |
| *Enterobacter cloacae* | *-* | ammonium sulfate (60-90%) precipitation, dialysis, ion-exchange chromatography and size exclusion chromatography | 105.07 IU/mg | (Husain, Sharma et al. 2016) |
| *Aspergillus fumigatus WL002* | *-* | Ultra filtration, Ammonium sulphate precipitation, 60%, anion exchange chromatography and gel filtration chromatography. | 355.33 ± 4.21 IU/mg | (Dutta, Ghosh et al. 2015) |
| *Bacillus licheniformis* RAM-8 | *-* | Ultrafiltration, Acetone precipitation, DEAE cellulose chromatography, Gel filtration. | 697.09 IU/mg | (Mahajan, Kumar et al. 2014) |
| *Bacillus licheniformis RAM-8* | *-* | Ultrafiltration, Acetone precipitation, ion-exchange chromatography & Gel filtration | 697.09 IU/mg | (Mahajan, Kumar et al. 2014) |
| *Bacillus subtilis B11−06* | pMA5 | two-step procedure including ammonium sulfate (80%) fractionation and hydrophobic interaction chromatography | 92.45 U/mg | (Jia, Xu et al. 2013) |
| *Streptomyces thermoluteus subsp. fuscus* NBRC 14270 (14270 ASNase) and *Streptomyces griseus* (SGR ASNase) | pGEM-T, pTONA5a | Filtration, gel filtration chromatography and dialysis. | 14270 ASNase-68.09 U/mg; SGR ASNase-48.89 U/mg | (Hatanaka, Usuki et al. 2011) |
| *Streptomyces gulbargensis* | *-* | ammonium sulfate fractionation (40-60%), gel filtration chromatography, ion exchange chromatography | 2053 IU/mg | (Amena, Vishalakshi et al. 2010) |
| *Bacillus sp.* | *-* | ammonium sulfate fractionation (70-100%), dialysis, ion exchange chromatography | 1.12 U/mg | (Moorthy, Ramalingam et al. 2010) |
| *Escherichia coli* BLR(DE3) | pelB leader sequence fused with inducible T7lac promoter | Ni-NTA affinity column chromatography, gel filtration, thrombin treatment and ultrafiltration. | 165 IU/mg | (Khushoo, Pal et al. 2005) |

**Table 3A: Acrylamide reduction table in fried food**

| **Source of the enzyme** | **Food matrix** | **Acrylamide reduction** | **Reference** |
| --- | --- | --- | --- |
| thermostable l-asparaginase from *Thermococcus zilligii AN1 TziAN1_1* | French fries | **80.5 %** | (Zuo, Zhang et al. 2015) |
| *Bacillus subtilis B11−06* | fried potato slices | **82%** | (Jia, Xu et al. 2013) |
| L-asparaginase (Novozymes, Denmark) | fried-dough pastry | upto **90%** | (Kukurová, Morales et al. 2009) |
| *A. Oryzae ZY087777* | French Fries, Sliced Potato Chips. | **60-85%** | (Hendriksen, Kornbrust et al. 2009) |

**Table 3B: Acrylamide reduction table in backed food**

| **Source of the enzyme** | **Food matrix** | **Acrylamide reduction** | **Reference** |
| --- | --- | --- | --- |
| *Cladosporium sp.* | Sweet Bread | **81%** | (Mohan Kumar, Shimray et al. 2014) |
| *A. Oryzae ZY087777* | Semisweet Biscuits, Ginger Biscuits, Crisp Bread. | **84%** | (Hendriksen, Kornbrust et al. 2009) |

**Reference**

Amena, S., et al. (2010). "Production, purification and characterization of L-asparaginase from Streptomyces gulbargensis." Brazilian journal of microbiology **41**: 173-178; <https://doi.org/110.1590/S1517-838220100001000025>.

Dias, F. F. G., et al. (2016). "Purification, characterization and antiproliferative activity of L-asparaginase from Aspergillus oryzae CCT 3940 with no glutaminase activity." Asian Pacific Journal of Tropical Biomedicine **6**(9): 785-794; <https://doi.org/710.1016/j.apjtb.2016.1007.1007>.

Dutta, S., et al. (2015). "L-asparaginase and L-glutaminase from Aspergillus fumigatus WL002: Production and some physicochemical properties." Applied biochemistry microbiology **51**: 425-431; <https://doi.org/410.1134/S0003683815040067>.

El-Naggar, N. E.-A., et al. (2018). "Purification, characterization and immunogenicity assessment of glutaminase free L-asparaginase from Streptomyces brollosae NEAE-115." BMC Pharmacology Toxicology **19**(1): 1-15; <https://doi.org/10.1186/s40360-40018-40242-40361>.

Hatanaka, T., et al. (2011). "Extracellular production and characterization of two Streptomyces L-asparaginases." Applied biochemistry biotechnology **163**: 836-844; <https://doi.org/810.1007/s12010-12010-19087-12019>.

Hendriksen, H. V., et al. (2009). "Evaluating the potential for enzymatic acrylamide mitigation in a range of food products using an asparaginase from Aspergillus oryzae." Journal of agricultural food chemistry **57**(10): 4168-4176; <https://doi.org/4110.1021/jf900174q>.

Husain, I., et al. (2016). "Purification and characterization of glutaminase free asparaginase from Enterobacter cloacae: in-vitro evaluation of cytotoxic potential against human myeloid leukemia HL-60 cells." PLoS One **11**(2): e0148877; <https://doi.org/0148810.0141371/journal.pone.0148877>.

Jia, M., et al. (2013). "Cloning, expression, and characterization of L-asparaginase from a newly isolated Bacillus subtilis B11–06." Journal of agricultural food chemistry **61**(39): 9428-9434; <https://doi.org/9410.1021/jf402636w>.

Khushoo, A., et al. (2005). "Optimization of extracellular production of recombinant asparaginase in Escherichia coli in shake-flask and bioreactor." Applied microbiology biotechnology **68**: 189-197; <https://doi.org/110.1007/s00253-00004-01867-00250>.

Kukurová, K., et al. (2009). "Effect of l‐asparaginase on acrylamide mitigation in a fried‐dough pastry model." Molecular Nutrition Food Research **53**(12): 1532-1539; <https://doi.org/1510.1002/mnfr.200800600>.

Kumar, S., et al. (2011). "Purification and characterization of glutaminase-free L-asparaginase from Pectobacterium carotovorum MTCC 1428." Bioresource technology **102**(2): 2077-2082; <https://doi.org/2010.1016/j.biortech.2010.2007.2114>.

Mahajan, R. V., et al. (2014). "Purification and characterization of a novel and robust L-asparaginase having low-glutaminase activity from Bacillus licheniformis: in vitro evaluation of anti-cancerous properties." PLoS One **9**(6): e99037; <https://doi.org/99010.91371/journal.pone.0099037>.

Mohan Kumar, N., et al. (2014). "Reduction of acrylamide formation in sweet bread with L-asparaginase treatment." Food Bioprocess Technology **7**(3): 741-748; <https://doi.org/710.1080/02652030701242558>.

Moorthy, V., et al. (2010). "Production, purification and characterisation of extracellular L-asparaginase from a soil isolate of Bacillus sp." African Journal of Microbiology Research **4**(18): 1862-1867; <https://www.internationalscholarsjournals.com/articles/production-purification-and-characterisation-of-extracellular-lasparaginase-from-a-soil-isolate-of-bacillus-sp>.

Nguyen, T. T. H., et al. (2016). "Optimization, purification and characterization of recombinant L-asparaginase II in Escherichia coli." African Journal of Biotechnology **15**(31): 1681-1691; <https://doi.org/1610.5897/AJB2016.15425>.

Rauh, V. and Y. Xiao (2022). "The shelf life of heat-treated dairy products." International Dairy Journal **125**: 105235, <https://doi.org/105210.101016/j.idairyj.102021.105235>.

Sidoruk, K., et al. (2011). "Creation of a producent, optimization of expression, and purification of recombinant Yersinia pseudotuberculosis L-asparaginase." Bulletin of experimental biology medicine **152**: 219-223; <https://doi.org/210.1007/s10517-10011-11493-10517>.

Upadhyay, A. K., et al. (2014). "Refolding and purification of recombinant L-asparaginase from inclusion bodies of E. coli into active tetrameric protein." Frontiers in Microbiology **5**: 486; <https://doi.org/410.3389/fmicb.2014.00486>.

Vala, A. K., et al. (2018). "Characterization of L-asparaginase from marine-derived Aspergillus niger AKV-MKBU, its antiproliferative activity and bench scale production using industrial waste." International journal of biological macromolecules **108**: 41-46; <https://doi.org/10.1016/j.ijbiomac.2017.1011.1114>.

Warangkar, S. C. and C. N. Khobragade (2010). "Purification, characterization, and effect of thiol compounds on activity of the Erwinia carotovora L-asparaginase." Enzyme research **2010**: <https://doi.org/10.4061/2010/165878>.

Zuo, S., et al. (2015). "Reduction of acrylamide level through blanching with treatment by an extremely thermostable L-asparaginase during French fries processing." Extremophiles **19**: 841-851; <https://doi.org/810.1007/s00792-00015-00763-00790>.
